# Breastmilk Feeding during the First 4 to 6 Months of Age and Childhood Disease Burden until 10 Years of Age

**DOI:** 10.3390/nu13082825

**Published:** 2021-08-17

**Authors:** Ju Hee Kim, Seung Won Lee, Jung Eun Lee, Eun Kyo Ha, Man Yong Han, Eun Lee

**Affiliations:** 1 Department of Pediatrics, Kangdong Sacred Heart Hospital, Hallym University Medical Center, Seoul 05355, Korea; 2004052@gmail.com; 2 Department of Data Science, Sejong University College of Software Convergence, Seoul 05006, Korea; lsw2920@gmail.com (S.W.L.); mrt6519@gmail.com (J.E.L.); 3 Department of Pediatrics, Hallym University Kangnam Sacred Heart Hospital, Seoul 07441, Korea; dmsry1@gmail.com; 4 Department of Pediatrics, Bundang CHA Medical Center, CHA University School of Medicine, Seongnam 13496, Korea; 5 Department of Pediatrics, Chonnam National University Hospital, Chonnam National University Medical School, Gwangju 61469, Korea

**Keywords:** breastfeeding, childhood diseases, benefit, hospitalization, overweight, obesity

## Abstract

Background: Breastfeeding is recommended due to its beneficial effects on human health. However, the effect of breastfeeding on health differs, resulting in various childhood diseases. Objective: Our purpose was to investigate the association between breastfeeding at least in the first 4 months and the subsequent development of 15 certainly defined childhood diseases until 10 years of age, the all-cause hospitalization rate and growth at 6–7 years of age. Methods: Participants included propensity-score matched 188,052 children born between January 2008 and December 2009, who were followed up till 10 years of age. Data were taken from the National Investigation of birth Cohort in Korea study 2008 database. Risk ratios were obtained using a modified Poisson regression and weighted risk differences using binomial regression. Results: Compared to formula feeding, breastfeeding was associated with decreased risks of febrile convulsion, attention deficit hyperactivity disorder and autism spectrum disorder, pneumonia, acute bronchiolitis, hypertrophic pyloric stenosis, asthma, all-cause hospitalization, overweight/obesity and short stature. Exclusive breastfeeding at 4 to 6 months of age had similar results to exclusive breastfeeding over 6 months of age. Conclusions: Breastfeeding in early infancy reduces the risk for various childhood diseases, all-cause hospitalization rate, obesity, and short stature during childhood.

## 1. Introduction 

Breastfeeding is the prerequisite for human evolution in terms of infant nutrition and subsequent health effects in later life. The health effects of breastfeeding results in various childhood diseases at different ages. Breastfeeding in early life is known to have beneficial effects on various childhood diseases, including infectious diseases such as respiratory tract infections [1], whereas its negative association with diseases such as intussusception has been identified [2,3]. In addition, the effect of breastfeeding on some diseases is inconclusive; for example, the effect of breastfeeding on malignancy is controversial [4,5]. Furthermore, no studies analyzing the association between breastfeeding and autoimmune diseases, including Hashimoto’s disease and alopecia areata, have been reported. As for the association of allergic diseases with breastfeeding, the recommendation strategies of breastfeeding and its duration for the prevention of allergic diseases, such as food allergy or atopic dermatitis have changed during the last decade according to the results of previous studies [6].

The inconclusive results on the effects of breastfeeding on childhood health might be partially attributable to the differences in the characteristics of the study population, follow-up duration, mechanisms underlying the association of breastfeeding and specific childhood disease and/or duration of breastfeeding [1,4,7,8,9,10]. Most of the previous studies on the health effect of breastfeeding had a small sample size, and studies including nationwide or worldwide cohorts are lacking. To confirm the results of the previous studies on the health effects of breastfeeding, studies on the health effect of breastfeeding in a larger study population are inevitable.

Although the World Health Organization (WHO) guidelines recommend exclusive breastfeeding for 6 months, the recommendation strategies of breastfeeding and its duration for the prevention of allergic diseases, including food allergy or atopic dermatitis, have changed in the last decade according to the results of previous studies [6]. Thus, more studies on the appropriate duration of exclusive breastfeeding are needed to improve children’s health.

Therefore, in the present study which includes a large number of children born between 2008 and 2009, we evaluated the association of breastfeeding in early life with common and important childhood diseases. The primary purpose of the present study was to investigate the association between breastfeeding and 15 childhood diseases in the nationwide cohort. The secondary purpose was to elucidate the association between breastfeeding and the risk of admission and need for intensive care during childhood. We also identified the association between exclusive breastfeeding in the first 4–6 months and over the first 6 months with childhood diseases.

## 2. Methods

### 2.1. Study Design and Setting

Information regarding the feeding type until the first 4 to 6 months of age was obtained from the National Investigation of birth Cohort in Korea study 2008 (NICKs-2008) in South Korea [11]. For the participants in the NICKs-2008, information regarding healthcare usage, including inpatient, outpatient, and pharmacy visits at healthcare facilities, until December 2017 was obtained from the National Health Insurance Service (NHIS) [11]. The Checklist of Recommendations based on Reporting of Studies Conducted using Observational Routinely-collected Health Data is described in Table S1.

### 2.2. Data Sources

From the NICKs-2008-NHIS database, information regarding the International Classification of Disease 10th Version (*ICD-10)* procedure and prescription codes at the time of inpatient, outpatient, and emergency department visits, as well as admission to the intensive care unit (ICU), were obtained (Figure S1). Height and weight were initially measured at 4 to 6 months of age and then four times annually during 30 to 71 months of age. Medical records of all of the participants in the present study were anonymized to ensure confidentiality. The study protocol was approved by the Institutional Review Board of the Korea National Institute for Bioethics Policy (P01-201603-21-005).

### 2.3. Study Population

**Figure 1:**
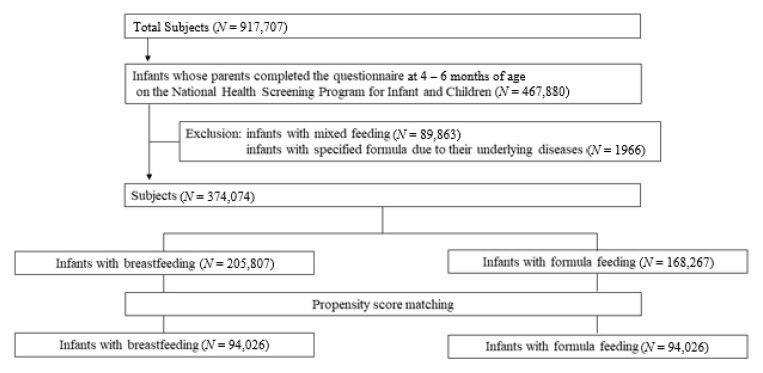
Diagram for cohort inclusion.

Among the 917,707 infants born between January 2008 and December 2009, 467,880 infants, whose data on feeding type until the first 4 to 6 months of age could be accessible, were enrolled in the present study. Infants who received mixed feeding (*n* = 89,863) and special milk due to underlying diseases (*n* = 1966) were excluded from the study. The flow diagram for the cohort is shown in Figure 1.

### 2.4. Exposure

The “exposure” was breastfeeding during the first 4 to 6 months of age. Questions on infant feeding practices were obtained using a parent-answered questionnaire at 4 to 6 months of age of the child. The question included “What do you feed your child usually?” The answers were as follows: (1) breastfeeding alone, (2) formula alone, and (3) mixed feeding with breastfeeding and formula. To classify children according to the duration of exclusive breastfeeding, questions on complementary feeding practices were obtained using a parent-answered questionnaires at 9 to 12 months of age. The question included “When did you start complementary feeding?” The answers were as follows: (1) before 4 months of age, (2) between 4 months to 6 months of age, (3) after 6 months of age, (4) not yet. Breastfed infants were further classified, (1) exclusively breastfed either in the first 4–6 months or (2) over the first 6 months.

### 2.5. Outcomes

The primary outcome was to investigate the associations between breastfeeding in the first 4 to 6 months and subsequent development of childhood diseases from 6 months till 10 years of age. Table S2 shows definitions of 15 certainly defined diseases and *19 ICD-10* codes based on these diseases. The diseases whose definitions have been validated using administrative data in a previous study are termed certainly defined diseases (Table S2). The secondary outcome was to investigate the association of breastfeeding in the first 4 to 6 months with the hospitalization rate as well as overweight (BMI for age z score ≥ 1.04)/obesity (BMI for age z score ≥ 1.64) [12] and −1.64 < height for age (HFA) z score ≤ −1.03/short stature (HFA z score ≤ −1.64) [13] at childhood. Children’s height and weight were measured at 6–7 years of age, and BMI was calculated as weight (kg) divided by height (m) squared.

### 2.6. Statistical Analyses

The propensity score (PS) was estimated using multivariable logistic regression with 26 covariates chosen a priori. The demographic and perinatal clinical variables in Table 1 and Table S3 in the supplement were considered as potential confounding factors for PS matching. Between-group differences in baseline characteristics were compared using standardized differences in both the unmatched and matched samples (differences > 10% were considered meaningful) [14]. Modified Poisson regressions were used to estimate crude risk ratios (RRs) and their 95% confidence intervals (CIs) of disease and all-cause hospitalization/mortality in breastfed children compared with formula milk-fed children. In addition, modified Poisson regressions were applied to evaluate crude RRs and their 95% CIs of disease and all-cause of hospitalization/mortality in breastfed children stratified into exclusive breastfeeding duration (4–6 months or over 6 months of age) compared with formula milk-fed children. The crude findings were further subjected to Bonferroni correction to account for multiple comparisons. We performed subgroup analysis by dividing the group based on gender (boys and girls) to analyze the association between exposure and the primary and secondary outcomes. To analyze the subgroup, analysis stratified into gender, binomial regression models with log function were used to estimate risk differences and their 95% CIs for 15 certainly defined diseases, all-cause hospitalization, and growth among breastfed children compared with those in formula milk-fed children [15]. In addition, to further robust our results, we additionally performed the analysis for the association of breastfeeding with subsequent development of childhood disease after 24 months of age.

All analyses were performed using SAS version 9.4 (SAS Institute Inc., Cary, NC, USA). Two-sided *p* < 0.05 was considered statistically significant.

## 3. Results

### 3.1. Study Population

The baseline socioeconomic characteristics of the study population are shown in Table 1. Before PS-matching, the standardized differences in age, height, weight, head circumference, prematurity, birth weight, and whether they have ever been fed anything other than breastmilk or formula milk until 4 months of age were >10%. After PS-matching, the breastfeeding and formula feeding groups were balanced for socioeconomic variables. In addition, the perinatal clinical condition and chromosome anomaly were balanced between both groups after PS-matching (Table S3).

### 3.2. Effect of Breastfeeding during the First 4 to 6 Months on Childhood Diseases

**Figure 2:**
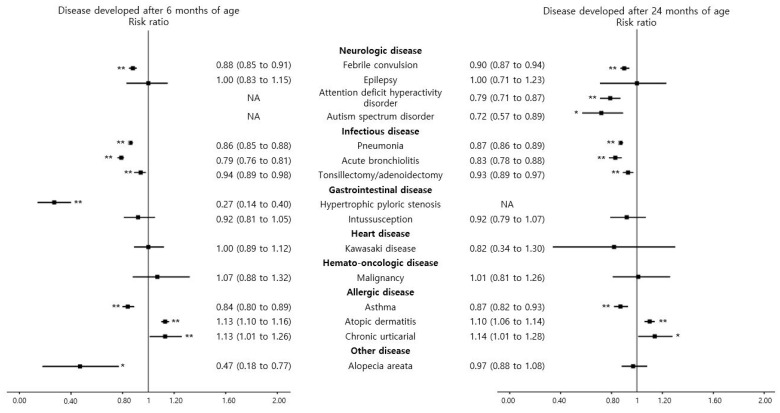
Risk of the prespecified diseases in children breastfed during the first 4 to 6 months of age compared with children fed formula milk during the first 4 to 6 months of age. Exceptionally, attention deficit hyperactive disorder included those diagnosed after age 4, and autism spectrum disorder included those diagnosed after 18 months. Black filled rectangles indicate the risk ratios, black lines indicate the 95% confidence intervals, and asterisks indicate *p* < 0.05. Double asterisks indicate *p* < 0.05 after Bonferroni correction for multiple comparisons.

The prevalence of each disease was comparable to that previously reported (Table S4). Figure 2 and Table S5 show the association of breastfeeding with 15 certainly defined diseases. The breastfeeding group had a significantly decreased risk of the diagnosis after 6 months of age for febrile convulsion (RR (95% CI), 0.88 (0.85 to 0.91)), as well as pneumonia (RR (95% CI), 0.86 (0.85 to 0.88)), acute bronchiolitis (RR (95% CI), 0.79 (0.76 to 0.81)), tonsillectomy or adenoidectomy (RR (95% CI), 0.94 (0.89 to 0.98)), hypertrophic pyloric stenosis (HPS) (RR (95% CI), 0.27 (0.14 to 0.40)), asthma (RR (95% CI), 0.84 (0.80 to 0.89)), and alopecia areata (RR (95% CI), 0.47 (0.18 to 0.77)) compared with the formula-feeding group. The breastfeeding group had a significantly decreased risk of diagnosis after 24 months of age of attention deficit hyperactive disorder (ADHD) (RR (95% CI), 0.79 (0.71 to 0.87)), and autism spectrum disorder (ASD) (RR (95% CI), 0.72 (0.57 to 0.89)). In addition, the associations between breastfeeding and diseases diagnosed after 24 months were similar to those after 6 months, except for alopecia areata.

However, the breastfeeding group had a higher risk of atopic dermatitis (RR (95% CI), 1.13 (1.10 to 1.16) and 1.10 (1.06 to 1.14), respectively) and chronic urticaria (RR (95% CI), 1.13 (1.01 to 1.26) and 1.14 (1.01 to 1.28), respectively) diagnosed after 6 months and 24 months of age than the formula-feeding group. In addition, the breastfeeding group had no association with epilepsy, intussusception, Kawasaki disease, and malignancy.

Regarding the diagnosis of defined diseases based on *ICD-10* based (Table S5), the breastfeeding group had a decreased risk for irritable bowel syndrome after 6 months and 24 months of age (RR (95% CI), 0.85 (0.80 to 0.90) and 0.85 (0.79 to 0.93), respectively), although they showed an increased risk of iron deficiency anemia diagnosed after 6 months and 24 months of age (RR (95% CI), 2.28 (2.21 to 2.35) and 1.47 (1.40 to 1.53), respectively), food allergy diagnosed after 6 months of age (RR (95% CI), 1.13 (1.05 to 1.23)), and congenital hypothyroidism diagnosed after 24 months of age (RR (95% CI), 1.49 (1.06 to 2.10)) compared with the formula feeding group. There was no statistical significance between the status of breastfeeding and gastrointestinal disease (acute pancreatitis and chronic viral (B, C) hepatitis), cardiovascular disease (arrythmia and acute myocarditis), idiopathic thrombocytopenic purpura, kidney disease (nephrotic syndrome, chronic kidney disease, and Henoch-Schönlein purpura), endocrine disease (goiter, Hashimoto’s disease, myasthenia gravis, and central precocious puberty), anaphylaxis, and juvenile rheumatoid arthritis.

### 3.3. Effect of Breastfeeding during the First 4 Months on All-Cause Hospitalization, ICU Admission, and Death Due to Childhood Diseases

**Figure 3:**
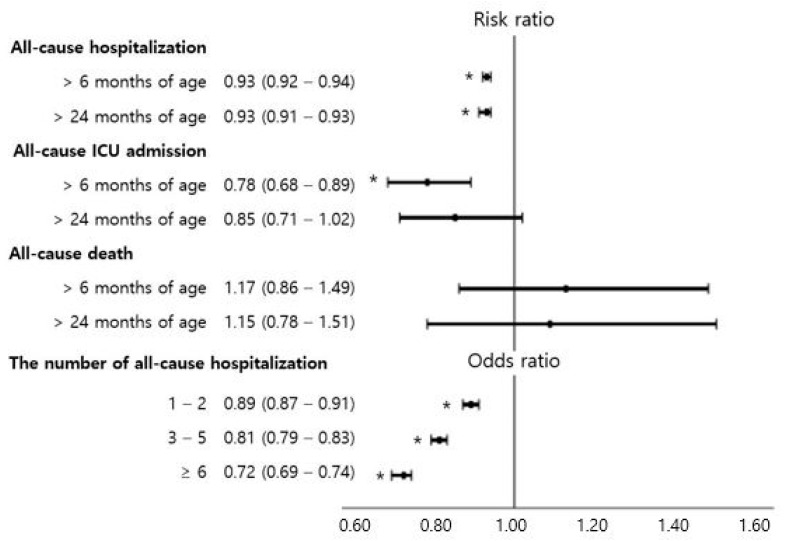
The risk of all-cause hospitalization and intensive care unit admission during childhood in children breastfed during their first 4 to 6 months of life compared with children fed formula milk in the first 4 to 6 months of age. Black filled rectangles indicate risk ratios or odds ratios, black lines indicate 95% confidence intervals, and asterisks indicate *p* < 0.05.

Figure 3 and Table S6 show the risks of hospitalization and death in breastfed children compared with those in formula-fed children. Breastfeeding in the first 4 to 6 months was associated with decreased risks of all-cause hospitalization after 6 months of age (RR (95% CI), 0.93 (0.92 to 0.94)) and after 24 months of age (RR (95% CI), 0.93 (0.91 to 0.93)). Furthermore, the more frequent the hospitalizations, the lower the risk of hospitalization of in breastfed children than in formula-fed children. Breastfeeding was related to the decreased risk of all-cause ICU admission after 6 months of age (RR (95% CI), 0.78 (0.68 to 0.89)). However, there was no significant association of breastfeeding in the first 4 to 6 months of age with all-cause death until 10 years of age.

### 3.4. Subgroup Analysis

**Figure 4:**
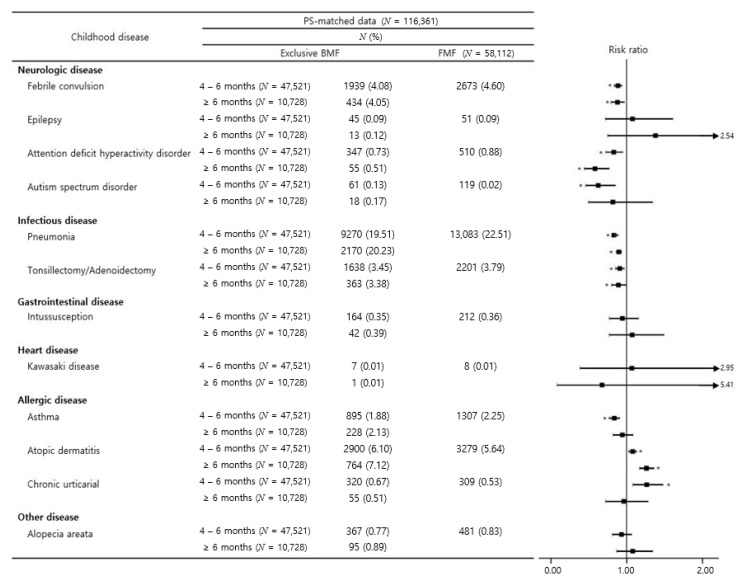
Risk for the pre-specified diseases in children grouped into exclusively breastfed during the first 4 to 6 months of age or over 6 months of age, compared with children fed formula milk during the first 4 to 6 months of age. Black filled rectangles indicate the risk ratios, black lines indicate the 95% confidence intervals, and asterisks indicate *p* < 0.05.

Figure 4 shows the subgroup analysis of certainly defined childhood disease by the duration of exclusive breastfeeding (4–6 months of age or over 6 months of age) compared to formula feeding. Both groups had similar effect on febrile seizure, ADHD, pneumonia, tonsillectomy/adenoidectomy, and atopic dermatitis. Although the group of exclusive breastfeeding over 6 months of age did not have a statistically significant effect on ASD and asthma, the group of exclusive breastfeeding over 6 months of age had statistically significant effect. In addition, both exclusive breastfeeding during 4 to 6 months of age and over 6 months decreased the all-cause hospitalization after 24 months of age (Table S7).

**Figure 5:**
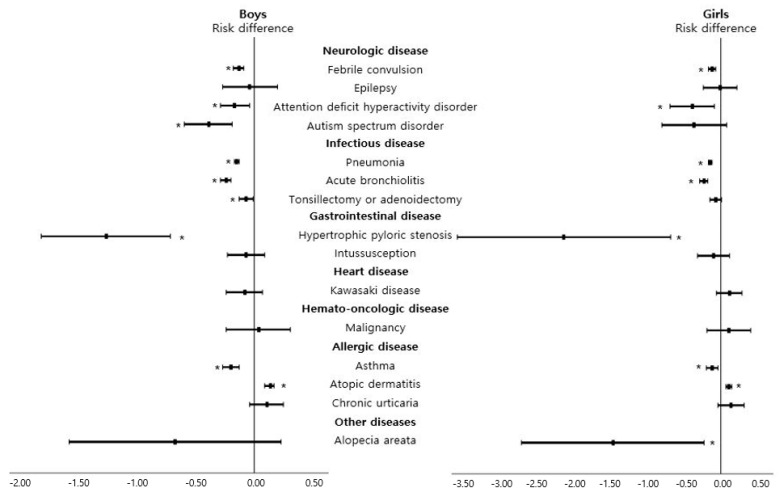
Subgroup analysis based on sex to analyze risk differences of the prespecified diseases in children breastfed during the first 4 to 6 months of age compared with children fed formula milk during the first 4 to 6 months of age. Black filled rectangles indicate risk ratios, black lines indicate 95% confidence intervals, and asterisks indicate *p* < 0.05.

Figure 5 and Table S8 show the association of breastfeeding with the subsequent development of 15 certainly defined diseases in boys and girls. Most results were consistent with our primary results, that is, the breastfeeding group showed a decreased risk of febrile convulsion, ADHD, ASD, pneumonia, acute bronchiolitis, HPS, and asthma and showed an increased risk of atopic dermatitis. In addition, the breastfeeding group showed less risk of all-cause hospitalization after 6 months of age than the formula-feeding group in both boys and girls (Table S9).

### 3.5. Association of Breastfeeding during the First 4 to 6 Months with Overweight/Obesity and Short Stature

**Figure 6:**
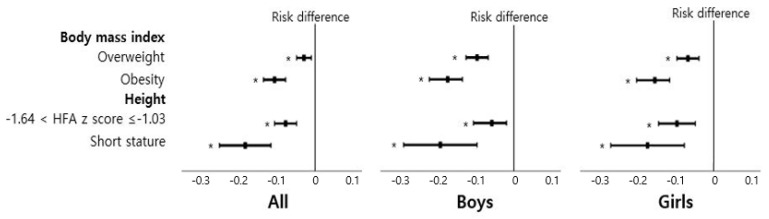
Association of breastfeeding during the first 4 to 6 months of age with child growth. Black filled rectangles indicate estimates of risk difference, black lines indicate 95% confidence intervals, and asterisks indicate *p* < 0.05. Overweight as BMI for age was defined as z score ≥ 1.03 and obesity as BMI for age z score ≥ 1.64. Short stature was defined as HFA z score ≤ −1.64.

Breastfeeding in the first 4 to 6 months of age was associated with the decreased risks of overweight (risk difference (95% CI), −0.03 (−0.05 to −0.01)) and obesity (risk difference (95% CI), −0.11 (−0.14 to −0.08)) at the age of 6–7 years (Figure 6 and Table S10). In addition, the breastfeeding group showed a significant association with −1.64 < HFA z score ≤ −1.03 (risk difference (95% CI), −0.08 (−0.11 to −0.05)) as well as short stature (risk difference (95% CI), −0.19 (−0.26 to −0.12)).

In subgroup analysis stratified by gender, the association of breastfeeding with overweight/obesity and short stature was consistent. In subgroup analysis into the duration of exclusively breastfeeding, only the group of exclusive breastfeeding during 4 to 6 months of age had significant associations with overweight, obesity, −1.64 < HFA z score ≤ −1.03, and short stature (Table S7).

## 4. Discussion

**Figure 7:**
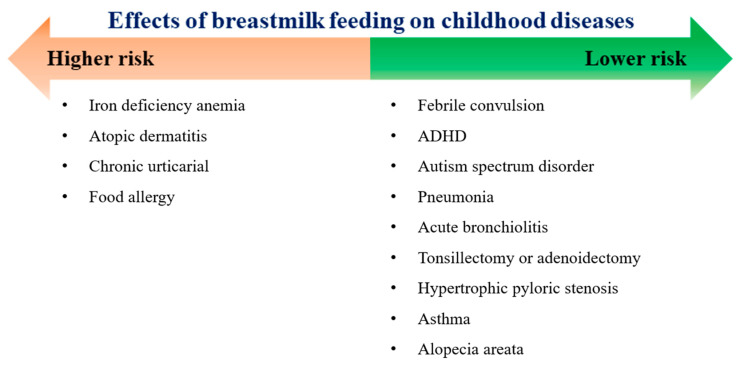
Association of breastmilk feeding in the first 4 to 6 months of age with health outcomes in childhood ADHD, attention deficit hyperactivity disorder.

We investigated the effect of breastfeeding an infant during the first 4 to 6 months of age on childhood diseases, hospitalization, ICU admission, and mortality, regardless of the causes, and growth until 10 years of age in a large population-based nationwide cohort. Breastfeeding in the first 4 to 6 months of age was associated with decreased risks of various diseases, including neurological diseases (febrile convulsion, ADHD, and ASD), respiratory infectious diseases (pneumonia and acute bronchiolitis), gastrointestinal diseases (hypertrophic pyloric stenosis and irritable bowel syndrome), asthma, and alopecia areata (Figure 7). Breastfeeding during the first 4 to 6 months of age decreased the risk of all-cause hospitalization and all-cause ICU admission until 10 years of age. Furthermore, breastfeeding was associated with a decreased risk for overweight and obesity at the age of 6–7 years.

We summarized the association of breastfeeding with childhood diseases in previous studies (Table 2 and Table S11). The previous studies have reported that breastfeeding decreases the risk of epilepsy [16], anxiety and depression [17], inflammatory bowel diseases [18], Kawasaki disease [19], chronic kidney diseases [20], Henoch-Schönlein purpura [21], central precocious puberty [22], anaphylaxis [23], and juvenile rheumatoid arthritis [24], whereas the present study showed no association of these diseases with breastfeeding in the first 4 to 6 months of age. In addition, the previous reports showed that breastfeeding increases the risk of intussusception [3], whereas the present study showed no association between them. To the best of our knowledge, there have been no studies on the association of breastfeeding with irritable bowel syndrome, alopecia areata, and all-cause admission to ICU; the present study showed that breastfeeding in the 4 to 6 months of age decreases the risk of these conditions. The present study showed no association of breastfeeding with acute pancreatitis, arrhythmia, acute myocarditis, idiopathic thrombocytopenic purpura, nephrotic syndrome, goiter, Hashimoto’s disease, myasthenia gravis, and hemolytic anemia; however, there have been no previous studies on these issues. Some studies showed a controversial association of breastfeeding with malignancy [4,5], atopic dermatitis [25], and food allergy [23,26]. However, the present study showed an increased risk of food allergy and atopic dermatitis and no association with malignancy in infants breastfed in their first 4 to 6 months of age. The discrepancy in the results of the previous studies and present study might be due to the sample size, characteristics of the study population, study design, and duration of breastfeeding; therefore, the application of the results needs to be individualized. 

In this world, nothing can replace breastmilk with regard to nutrients and emotional connection. Breastmilk contains diverse macronutrients, micronutrients, microbial communities, hormones, growth factors, and microRNAs [43,44]. The complex interaction of these factors influence the composition and balance of the intestinal microbiome and their metabolites, thereby affecting the diverse disease susceptibility in later life [45]. The immune system developed in early life lasts beyond infancy, thereby affecting health even during childhood. Although breastfeeding is known to be beneficial in decreasing the burden of childhood diseases, studies on the individualized mechanism underlying each childhood disease is lacking; therefore, future studies are needed to identify the mechanisms underlying the beneficial effects of breastfeeding in each disease and to promote the beneficial role of breastfeeding in improving children’s health. 

The increased risk of childhood diseases, such as iron deficiency anemia in infants with breastfeeding might be associated with deficient components in breastmilk. Breast milk does not carry enough iron to meet estimated needs [46], and the content of minerals in breast milk, including iron, is not significantly affected by maternal nutritional and diet status [47]. The increased risk of food allergy in breastfed infants might be associated with restriction of formula feeding in infants with cow’s milk allergy, which is the most common cause of food allergy in early life [48]. Studies regarding the effect of supplementation of absent components in breast milk on childhood health are required, to improve childhood health among breastfed infants.

The association of breastmilk feeding with atopic dermatitis is inconclusive [10]. A recent meta-study, including 27 studies, showed no significant association, with a protective effect of breastfeeding on atopic dermatitis in cohorts with atopic heredity alone [10]. Multifactorial factors, including a family history of allergic diseases, skin barrier dysfunction, and immune dysregulation, play a role in the development of atopic dermatitis; thus, characteristics of the study population might affect the association of breastfeeding and atopic dermatitis.

In a subgroup analysis of the duration of exclusively breastfeeding, we identified both groups of exclusive breastfeeding during 4 to 6 months of age and over 6 months of age had similar effect on most childhood disease and all-cause hospitalization compared to formula feeding. However, only exclusive breastfeeding during the first 4–6 months had beneficial associations with ASD, asthma, overweight, obesity, and short stature, while exclusive breastfeeding over 6 months was not. Until now, there has been no consensus on when to start complementary feeding. WHO guidelines recommend exclusive breastfeeding for first 6 months [49], but ESPGHAN recommends that complementary feeding should be initiated between 17 weeks and 26 weeks of age [50]. However, our results were not sufficient to conclude the superiority of the introduction of complementary feeding at 4 to 6 months of age or after 6 months. Therefore, further study must be needed to confirm a novel timing to initiate complementary feeding.

The results of the present study are significant in that this study has identified the overall childhood health effect of breastfeeding in the first 4 to 6 months of age in the general population-based nationwide cohort covering > 90% of children born during 2008–2009 in South Korea combined with national claims, which also covers > 90% of the total population. In addition, this study included the health effects of breastfeeding during the important period of childhood for a long period follow-up of 10 years. To reduce bias, we performed PS-matching analysis in the national health insurance service claims data and adjusted for important confounding factors, including socioeconomic status and perinatal factors, which have a significant impact on children’s health [8,9]. Therefore, the results of the present study can confirm the results of the previous studies (Table 2 and Table S10) on the health effects of breastfeeding, performed in the small sample-sized population [1,4,8,10,40], by improving the power of confidence resulting from the large numbers of participants. In addition, we added information regarding the effects of breastfeeding on childhood diseases that have not been identified, such as acute pancreatitis and nephrotic syndrome. Therefore, the results of the present study would be helpful in establishing policies regarding recommendation guidelines for breastfeeding in early life.

Nevertheless, the present study has some limitations. First, the ascertainment of childhood diseases was identified using *ICD-10* codes at the time of outpatient, inpatient, or emergency department visits, which may have led to overestimation of the diagnosis. However, the prevalence or incidence of each disease in the present study was comparable to that reported in the previous studies. Furthermore, to improve the accuracy of each disease definition, we combined the number of claims during specific periods in some chronic diseases, which required recurrent hospital visits, in conjunction with *ICD-10* codes. In addition, if certain diseases required disease-specific procedure or medication, we combined the *ICD-10* codes with disease-specific procedure or prescription codes to improve the accuracy of the specific diseases. Second, information regarding the duration of breastfeeding and disease severity was lacking. Thus, the effect of the duration of breastfeeding on diverse childhood diseases could not be analyzed in the present study. Third, this study was an observational study, because of which causality cannot be established. In addition, information on the possible confounding factors, such as immunization and nutrition, could not be obtained, although the confounding factors might be limited to specific diseases. The health effects of breastfeeding may be affected by income levels [9], and the present study was performed in a middle-income country, which limits the generalization of the results of the present study. Despite these limitations, this study itself has significance in that the present study has identified the diverse health effects of breastfeeding during the first 4 to 6 months on the burden of childhood diseases.

## 5. Conclusions

Breastfeeding in early life has beneficial effects against the subsequent development of various childhood diseases, including obesity, as well as risk of hospitalization rate during childhood. In addition, this study shows that exclusive breastfeeding in the first 4–6 months was not inferior to exclusive breastfeeding in the first 6 months in decreasing the risk of childhood diseases, hospitalization, obesity and short stature compared to formula feeding. Our results will improve understanding regarding the health benefits of breastfeeding in children, thereby contributing in the establishment of breastfeeding guidelines to improve global childhood health.

## Figures and Tables

**Table 1:** Baseline socioeconomic characteristics of the participants ^a^.

Sociodemographic Characteristics	All Data (*n* = 374,074)			PS-Matched Data (*n* = 188,052) ^b^		
	*N* (%) ^c^		Standardized Difference % ^f^	*N* (%) ^c^		Standardized Difference % ^f^
	Breast Feeding ^d^ (*N* = 205,807)	Formula Feeding ^e^ (*N* = 168,267)		Breast Feeding ^d^ (*N* = 94,026)	Formula Feeding ^e^ (*N* = 94,026)	
Boys	101,611 (49.37)	89,571 (53.23)	8.0	47,985 (51.0)	48,709 (51.8)	1.5
Age, days ^g,h^	168.04 (29.74)	170.93 (29.92)	16.4	181.17 (26.25)	181.43 (26.61)	1.0
Height, m ^g,h^	0.67 (0.03)	0.68 (0.03)	14.0	0.68 (0.03)	0.68 (0.03)	4.5
Weight, kg ^g,h^	8.08 (1.01)	8.21 (1.03)	14.2	8.31 (1.01)	8.34 (0.99)	3.3
HC, cm ^g,h^	42.65 (1.50)	42.87 (1.55)	14.0	43.06 (1.46)	43.11 (1.47)	3.1
BMI ^g,h^	17.89 (1.67)	17.87 (1.64)	0.5	17.88 (1.66)	17.89 (1.62)	0.5
Prematurity	4722 (2.29)	8818 (5.24)	14.5	4035 (4.30)	4000 (4.30)	0.2
Birth weight, kg ^h^	3.24 (0.41)	3.16 (0.47)	16.4	3.22(0.40)	3.21(0.43)	1.8
Have your child ever fed anything other than breastmilk or formula until 4 months of age? ^i^						
yes	106,211 (51.78)	104,545 (62.28)	18.7	86,625 (92.1)	87,822 (93.4)	4.6
no	98,925 (48.22)	63,327 (37.72)		7401 (7.9)	6204 (6.6)	
When did your child firstly fed anything other than breastmilk or formula? ^g^						
<4 months	7310 (6.15)	7201 (6.51)	1.5	5930 (6.3)	5932 (6.3)	0.0
≥4 months	111,553 (93.85)	103,488 (93.49)		88,096 (93.4)	88,094 (93.7)	
Residential area at birth ^j^						
Seoul	53,032 (26.00)	38,836 (23.30)	4.7	24,224 (25.8)	23,311 (24.8)	0.7
Metropolitan	46,867 (22.98)	40,092 (24.05)		21,616 (23.0)	22,921 (24.4)	
City	80,996 (39.31)	67,580 (40.54)		37,923 (40.3)	37,402 (39.8)	
Rural	23,069 (11.31)	20,182 (12.11)		10,263 (10.9)	10,392 (11.1)	
Income quintile ^k^						
1 (Lowest)	15,399 (7.75)	13,627 (8.37)	2.8	7550 (8.0)	7477 (8.0)	0.3
2	29,654 (14.93)	25,194 (15.48)		14,241 (15.2)	14,490 (15.4)	
3 (Middle)	54,397 (27.38)	45,192 (27.77)		25,989 (27.6)	26,011 (27.7)	
4	65,727 (33.09)	52,256 (32.12)		30,662 (32.6)	30,525 (32.5)	
5 (Highest)	33,464 (16.85)	26,446 (16.25)		15,584 (16.6)	15,523 (16.5)	

Abbreviations, PS, propensity score; N, number; HC, head circumference; BMI, body mass index; SD, standard deviation. ^a^ Unless otherwise specified, baseline characteristics were assessed on enrolled day at 4 to 6 months of age. ^b^ Propensity score matching (1:1) was performed to reduce bias for the selection of the comparison group. Matching was performed by Mahalanobis algorithm with a caliper of 0.01 using multivariable logistic regression with 23 previously chosen covariates. ^c^ Results are reported as N (%), unless otherwise indicated. ^d^ The breastfeeding groups comprised children who have been breastfed until the first 4 to 6 months of life. ^e^ As the reference group, the formula feeding group comprises children who have been fed formula milk until the first 4 to 6 months of life. ^f^ Differences > 10% were interpreted as a meaningful difference. All standardized differences of cohort values were <0.05. ^g^ These were measured at 4 to 6 months of birth. ^h^ Results are reported as means (SDs). ^i^ obtained by the first national health screening program of infants and children at 4 to 6 months of birth. Missing data in all data; breastfeeding group = 671, formula feeding group = 395. ^j^ Metropolitan areas were defined as six metropolitan cities (Busan, Incheon, Gwangju, Daejeon, Daegu, and Ulsan), urban areas as cities, and rural areas as non-city areas. Missing data in all data; breastfeeding group = 1843, formula feeding group = 1577. ^k^ Income status was categorized into quintiles of insurance premium at birth. Missing data of all data; control group = 7166, infantile colic group = 5552.

**Table 2:** Comparisons of the results of the previous studies on the association of breastfeeding with childhood diseases in children.

Childhood Diseases	Previous Studies ^b^	Present Study ^b^
Neurological diseases		
Febrile convulsion	↓ [27,28]	↓
Epilepsy	↓ [16]	No association
Attention deficit hyperactivity disorder	↓ [29,30]	↓
Autism spectrum disorder	↓ [31,32]	↓
Infectious diseases		
Pneumonia	↓ [33,34]	↓
Acute bronchiolitis	↓ [35]	↓
Tonsillectomy or adenoidectomy	NA	↓
Gastrointestinal diseases		
Hypertrophic pyloric stenosis	↓ [36]	↓
Intussusception	↑ [2]	No association
Cardiovascular diseases		
Kawasaki disease	↓ [19]	No association
Hemato-oncologic diseases		
Malignancy	Controversial [4,5]	No association
Allergic diseases		
Asthma	↓ [9,37]	↓
Atopic dermatitis	Controversial [25]	↑
Chronic urticaria	NA	↑
Other diseases		
Alopecia areata	NA	↓
Number of prescription of antibiotics during childhood	↓ [38]	↓
Number of admissions during childhood	↓ [39,40]	↓
Admission to intensive care unit during childhood	NA	↓
Childhood obesity ^a^	↓ [41,42]	↓

^a^ Obesity was defined as BMI for age z score ≥ 1.64. ^b^ ↓ means that breastfeeding shows the protective effects on each disease. ↑ means that breastfeeding shows the increased associations with each disease. NA: not applicable—no research has been identified.

## Data Availability

This study was based on the National Health Claims Database (NHIS-2019-1-560) established by the NHIS of the Republic of Korea. Applications for using NHIS data are be reviewed by the Inquiry Committee of Research Support; if the application is approved, raw data is provided to the applicant for a fee. We cannot provide access to the data, analytic methods, and research materials to other researchers because of the intellectual property rights of this database that is owned by the National Health Insurance Corporation. However, investigators who wish to reproduce our results or replicate the procedure can be used in the database, which is open for research purposes (https://nhiss.nhis.or.kr/ accessed on 14 August 2021).

## Supplementary Materials

The following are available online at https://www.mdpi.com/article/10.3390/nu13082825/s1, Figure S1: Present study design. Association of breastmilk feeding in the first 4 to 6 months of age with health outcomes in childhood. Table S1: Checklist of Recommendations for Reporting of Observational Studies Using the Reporting of Studies Conducted Using Observational Routinely Collected Health Data (RECORD) Guidelines. Table S2: Definitions of childhood diseases shown in the present study. Table S3: Perinatal clinical conditions of participantsa. Table S4: Comparisons of the prevalence of childhood diseases reported in other studies and that in the present study. Table S5: Risk for various childhood diseases in breastfed children during the first 4 to 6 months of age compared with formula fed children. Table S6: The risk of all-cause hospitalization and intensive care unit admission during childhood in children breastfed in their first 4 to 6 months of age, when formula fed infants during the first 4 to 6 months of age were considered reference group. Table S7: The risk of all-cause hospitalization and intensive care unit admission after 24 months of age and their growth in children stratified into the duration of exclusive breastfeeding (first 4 to 6 months of age or over 6 months of age), when formula fed infants during the first 4 to 6 months of age were considered reference group. Table S8: Gender differences of risk of childhood diseases in children who were breastfed in the first 4 to 6 months of age with children fed formula milk as the reference group. Table S9: Risk of all-cause admission in children who were breastfed in the first 4–6 months of age when stratified by gender. Table S10: Effect of breastfeeding during the first 4 to 6 months of age on body weight and height during childhood. Table S11: Comparisons of the results of the previous studies on association between breastfeeding and *ICD-10* code-based childhood diseases.

## Abbreviations

NICKs-2008, National Investigation of birth Cohort in Korea study 2008; NHIS, National Health Insurance Service; *ICD-10*, International Classification of Disease 10th Version; ICU, Intensive care unit; BMI, Body mass index; PS, propensity score; RR, Risk ratios; CI, confidence interval; OR, odds ratios; ADHD, Attention deficit hyperactivity disorder; ASD, Autism spectrum disorder; HPS, Hypertrophic pyloric stenosis; HFA, Height for age.
