# Supplementary material for: Breastmilk Feeding during the First 4 to 6 Months of Age and Childhood Disease Burden until 10 Years of Age
Source: Nutrients. 2021 Aug 17;13(8):2825. doi: 10.3390/nu13082825 (PMC8400284; doi:10.3390/nu13082825)
Supplement: Supplementary file 1 [file nutrients-13-02825-s001.zip › nutrients-1329696-supplementary.pdf]

Figure S1. Present study design

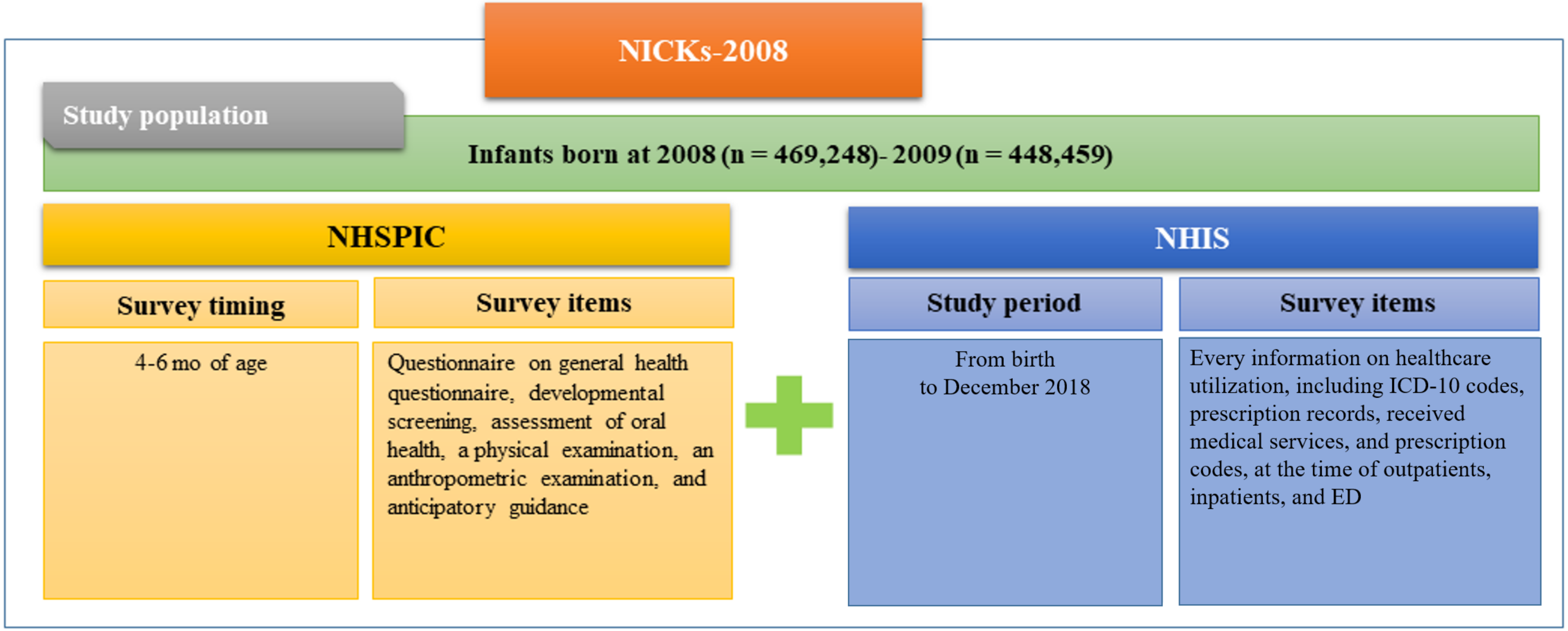

ED, emergency department; ICD-10, International Classification of Disease, 10th Version; NHIS, National Health Insurance Service; NHSPIC, National Health Screening Program for Infants and Children; NICKs-2008, National Investigation of birth Cohort in Korea study 2008

Table S1. Checklist of Recommendations for Reporting of Observational Studies Using the Reporting of Studies Conducted Using Observational Routinely Collected Health Data (RECORD) Guidelines

	Item No	Recommendation	Reported
Title and abstract	1	(a) Indicate the study's design with a commonly used term in the title or the abstract	Abstract
		(b) Provide in the abstract an informative and balanced summary of what was done and what was found	Abstract
**Introduction**			
Background/rationale	2	Explain the scientific background and rationale for the investigation being reported	Introduction
Objectives	3	State specific objectives, including any prespecified hypotheses	Introduction
**Methods**			
Study design	4	Present key elements of study design early in the paper	Methods- Study Design and Setting
Setting	5	Describe the setting, locations, and relevant dates, including periods of recruitment, exposure, follow-up, and data collection	Methods- Study Design and Setting
Participants	6	(a) Give the eligibility criteria, and the sources and methods of selection of participants. Describe methods of follow-up	Methods- Study Design and Setting; Figure 1
		(b) For matched studies, give matching criteria and number of exposed and unexposed	Methods- eFigure 1, Table 1, and eTable 3
Variables	7	Clearly define all outcomes, exposures, predictors, potential confounders, and effect modifiers. Give diagnostic criteria, if applicable	Methods- exposure and outcome; eTables 2 and 3
Data sources/measurement	8	For each variable of interest, give sources of data and details of methods of assessment (measurement). Describe comparability of assessment methods if there is more than one group	Methods- Study Design and Setting, Data Source, and Statistical Analyses; eTable 2
Bias	9	Describe any efforts to address potential sources of bias	Methods: Statistical Analyses, Table 1 and eTable 3
Study size	10	Explain how the study size was arrived at	Methods- Study Design and Setting; Figure 1

Quantitative variables	11	Explain how quantitative variables were handled in the analyses. If applicable, describe which groupings were chosen and why	Methods- Study Design and Setting; Figure 1 and eFigure 1
Statistical methods	12	(a) Describe all statistical methods, including those used to control for confounding	Methods: Statistical Analyses
		(b) Describe any methods used to examine subgroups and interactions	Methods: Statistical Analyses
		(c) Explain how missing data were addressed	Methods: Statistical Analyses
		(d) If applicable, explain how loss to follow-up was addressed	Not applicable
		(e) Describe any sensitivity analyses	Methods: Statistical Analyses
**Results**			
Participants	13	(a) Report numbers of individuals at each stage of study--e.g. numbers potentially eligible, examined for eligibility, confirmed eligible, included in the study, completing follow-up, and analyzed	Figures 1 and eFigure 1
		(b) Give reasons for non-participation at each stage	Figure 1
		(c) Consider use of a flow diagram	Figure 1
Descriptive data	14	(a) Give characteristics of study participants (e.g. demographic, clinical, social) and information on exposures and potential confounders	Method, Results: Study Population; Table 1
		(b) Indicate number of participants with missing data for each variable of interest	Table 1
		(c) Summarize follow-up time (e.g. average and total amount)	Methods: Study Design and Setting; Figure 1
Outcome data	15	Report numbers of outcome events or summary measures over time	Table 5,6,8 and 9
Main results	16	(a) Give unadjusted estimates and, if applicable, confounder-adjusted estimates and their precision (e.g. 95% confidence interval). Make clear which confounders were adjusted for and why they were included	Methods: Statistical Analyses, Tables 5-9
		(b) Report category boundaries when continuous variables were categorized	Not applicable
		(c) If relevant, consider translating estimates of relative risk into absolute risk for a meaningful time period	Results
Other analyses	17	Report other analyses done--e.g. analyses of subgroups and interactions, and sensitivity	Figures 2-5 and eTables

		analyses	5-9
**Discussion**			
Key result	18	Summarize key results with reference to study objectives	Discussion
Limitation	19	Discuss limitations of the study, taking into account sources of potential bias or imprecision. Discuss both direction and magnitude of any potential bias	Discussion
Interpretation	20	Give a cautious overall interpretation of results considering objectives, limitations, multiplicity of analyses, results from similar studies, and other relevant evidence	Discussion
Generalizability	21	Discuss the generalizability (external validity) of the study results	Discussion
Other information			
Funding	22	Give the source of funding and the role of the funders for the present study and, if applicable, for the original study on which the present article is based	Article information

Table S2. Definitions of childhood diseases shown in the present study

Variables	Definition	References
Certainly defined childhood diseases		
Neurologic diseases		
Febrile convulsion	ICD-10 code R56	[1, 2]
Epilepsy	ICD-10 codes G40 or G41.X and ≥ 2 claims of antiepileptic medications within 6 months	[2]
ADHD	ICD-10 code F90.0 and ≥2 claims within 6 months after 4 years old	[3]
Autism spectrum disorder	ICD-10 codes (F84.0, F84.1, or F84.9) with ≥ 2 claims within 6 months after the age of 18 months	[4]
Infectious diseases		
Pneumonia	ICD-10 codes (J12.0-J18.0) at admission	[5]
Acute bronchiolitis	ICD-10 code (J21.x) at admission	[6]
Tonsillar and/or adenoid hypertrophy	Tonsillar and/or adenoid hypertrophy requiring tonsillectomy and/or adenoidectomy	
Gastrointestinal diseases		
Hypertrophic pyloric stenosis	ICD-10 code (Q40.0) with pyloromyotomy	[7]
Intussusception	ICD-10 code (K56.1) with air reduction or manual reduction	[8]
Heart diseases		
Kawasaki disease	ICD-10 code (M30.3) with the use of intravenous immunoglobulin and aspirin (≥ 25 days)	[9]
Allergic diseases		
Asthma	ICD-10 codes (J45.X, J46.X) at admission	[10]
Atopic dermatitis	ICD-10 codes (J30.1-J30.4) with ≥ 5 claims and the use of topical corticosteroids ≥2 times	[11]
Chronic urticaria	ICD-10 codes (L50.1, L50.8, L50.9) with ≥ 2 claims of antihistamines within 6 weeks	[12]
Other disease		
Alopecia areata	ICD-10 code (L63.X) with ≥ 3 claims by dermatologists	[13]
ICD-10 code-based definition of childhood diseases		
Gastrointestinal diseases		
Irritable bowel syndrome	ICD-10 code (K58.X)	[14]
Acute pancreatitis	ICD-10 code (K85) at admission	[15, 16]
Chronic viral (B, C) hepatitis	ICD-10 codes (B18.1, 18.2)	[17, 18]

Heart diseases		
Arrhythmia	ICD-10 codes (I47.x-I49.x) at admission	[19]
Acute myocarditis	ICD-10 codes (I40.x-I41.x) at admission	[20]
Hemato-oncologic diseases		
Idiopathic thrombocytopenic purpura	ICD-10 code (D693.x)	[21, 22]
Iron deficiency anemia	ICD-10 code (D50.9)	
Hemolytic anemia	ICD10 code (D59)	[23]
Kidney diseases		
Nephrotic syndrome	ICD-10 codes (N05.x)	[24]
Chronic kidney diseases	ICD-10 codes (N18.x)	[25]
Henoch-Schönlein purpura	ICD-10 code (M36)	[26]
Endocrine diseases		
Congenital hypothyroidism	ICD-10 codes (E03.0, E03.1)	[27]
Goiter	ICD-10 code (E04.9X)	[28]
Hashimoto's disease	ICD-10 codes (E06.3, E06.9)	[29]
Myasthenia gravis	ICD-10 code (G70.0)	[30]
Central precocious puberty	ICD-10 code (E30.1)	[31]
Allergic diseases		
Anaphylaxis	ICD-10 codes (T78.0, T78.2) at admission or ED visit	[32]
Food allergy	ICD-10 code (T78.10)	[33]
Rheumatic diseases		
Juvenile rheumatoid arthritis	ICD10 code (M08.X)	[34]

ADHD, attention deficit hyperactivity disorder; ED, emergency department; ICD, International Classification of Diseases 10th revision.

Table S3. Perinatal clinical conditions of participants [a]

Variables, N (%)	All data (N = 374,074)			PS-matched data (N = 188,052) [b]		
	Breastfeeding[c] (N = 205,807)	Formula Feeding[d] (N = 168,267)	Standardized Difference %[f]	Breastfeeding[c] (N = 94,026)	Formula Feeding[d] (N = 94,026)	Standardized Difference %[f]
Fetus and newborn affected by maternal factors and by complications of pregnancy, labour and delivery	4,551 (2.21)	5,047 (3.00)	3.9	2,245 (2.40)	2,260 (2.40)	0.1
Disorders related to length of gestation and fetal growth	4,781 (2.32)	8,268 (4.91)	12.4	2,307 (2.50)	2,280 (2.40)	0.2
Birth trauma	1,981 (0.96)	1,656 (0.98)	0.7	894 (1.00)	911 (1.00)	0.2
Respiratory and cardiovascular disorders specific to the perinatal period	11,011 (5.35)	11,742 (6.98)	6.0	5,188 (5.50)	5,287 (5.60)	0.4
Infections specific to the perinatal period	30,551 (14.84)	27,205 (16.17)	3.4	14,147 (15.10)	14,283 (15.20)	0.4
Hemorrhagic and hematological disorders of fetus and newborn	73,480 (35.70)	53,178 (31.60)	8.6	30,905 (32.90)	29,742 (31.60)	2.6
Transitory endocrine and metabolic disorders specific to fetus and newborn	7,866 (3.82)	9,173 (5.45)	6.6	3,761 (4.00)	3,801 (4.00)	0.2
Digestive system disorders of fetus and newborn	6,164 (3.00)	5,432 (3.23)	0.8	2,816 (3.00)	2,792 (3.00)	0.1
Conditions involving the integument and temperature regulation of fetus and newborn	7,642 (3.71)	6,373 (3.79)	0.3	3,515 (3.70)	3,535 (3.80)	0.1
Congenital malformations, deformations, and other disorders originating in the perinatal period	12,587 (6.12)	11,498 (6.83)	2.8	5,825 (6.20)	5,884 (6.30)	0.3
Chromosomal abnormality	20,748 (10.08)	19,355 (11.50)	4.5	9,610 (10.20)	9,738 (10.40)	0.4

Abbreviations, N, number.

[a] Unless otherwise specified, all of perinatal clinical condition was assessed at birth.

[b] Propensity score matching (1:1) was performed to reduce bias for the selection of the comparison group. Matching was performed by Mahalanobis algorithm with a caliper of 0.01 using multivariable logistic regression with 23 previously chosen covariates.

[c] The breastfeeding group comprises children who have been breastfed until the first 4 to 6 months of age.

[d] As the reference group, the formula feeding group comprised children who have been fed formula milk until the 4 to 6 months of age.

[f] Differences > 10% were interpreted as a meaningful difference. All standardized differences of cohort values were < 0.05.

Table S4. Comparisons of the prevalence of childhood diseases reported in other studies and that in the present study

Childhood diseases	Prevalence in other studies	Prevalence in the present studies, n (%), total n = 374,074	References
Certainly defined childhood diseases			
Neurologic diseases			
Febrile convulsion	6.9% in South Korea	23,591 (6.3)	35
Epilepsy	0.66% in Norway	1,246 (0.3)	36
Attention deficit hyperactivity disorder	1.7% school-aged children in South Korea, 5% in meta-analysis	3,211 (0.9)	37, 38
Autism spectrum disorder	0.75-2.64% in South Korea	606 (0.2)	39
Infectious diseases			
Pneumonia	38.2-63.4% in children	97,343 (26.0)	40
Acute bronchiolitis	17.9-13.5/1000 person-years in children in the United States	25,160 (6.7)	41
Tonsillar or adenoid hypertrophy	77 (3.3%)/2,248 in 6-11-year-old children in South Korea	13,152 (3.5)	42
Gastrointestinal diseases			
Hypertrophic pyloric stenosis	0.39/1000 live births incidence	185 (0.0)	43
Intussusception	Incidence, 22.2 (95% CI, 13.9-33.7)/100,000 infants in Fiji	1,727 (0.5)	44
Heart diseases			
Kawasaki disease	17.5-20.8/100,000 children < 5 years in US	2,251 (0.6)	45
Hemato-oncologic diseases			
Malignancy	124.0-140.6/1,000,000/year in children aged 0-14 years (worldwide)	773 (0.2)	46
Allergic diseases			
Asthma	18.4-23.0/10,000/year	12,004 (3.2)	47
Atopic dermatitis	13.4-27.0% in children aged 6-7 years and those aged 12-13 years in South Korea	39,638 (10.6)	48
Chronic urticaria	2,256.5/100,000/year in South Korea	2,441 (0.7)	49
Other diseases			
Alopecia areata	0.57–2.1 from global incidence data	75 (0.0)	50

ICD-10 code-based definition of childhood diseases			
Gastrointestinal diseases			
Irritable bowel syndrome	22.0-35.5% in children in Canada	8,728 (2.3)	[51, 52]
Acute pancreatitis	1.9-2.2/100,000 person in children in the United States	47 (0.0)	[53]
Chronic viral (B, C) hepatitis	Global prevalence of chronic B hepatitis, 1.3%; chronic C hepatitis, 0.15%	48 (0.0)	[54, 55]
Heart diseases			
Arrhythmia	24.4/100,000 live births in infants	135 (0.0)	[56]
Acute myocarditis	1-2/100,000 children	20 (0.0)	[57]
Hemato-oncologic diseases			
Idiopathic thrombocytopenic purpura	1.9-6.4/100,000/year in children	885 (0.2)	[58]
Iron deficiency anemia	7.8% in infants aged 12 months	36,194 (9.7)	[59]
Hemolytic anemia	1/100,000 in children	195 (0.1)	[60]
Kidney diseases			
Nephrotic syndrome	2-7/100,000 in children	68 (0.0)	[61]
Chronic kidney diseases	18.5-59.3/1,000,000 in children	7 (0.0)	[62]
Henoch-Schönlein purpura	6-22/100,000/year in children	2,446 (0.7)	[63]
Endocrine diseases			
Congenital hypothyroidism	5.02/1000 births	336 (0.1)	[64]
Goiter	No information in the general population-based studies	610 (0.2)	
Hashimoto's disease	3.5-5/1000 in women and 0.6-1/1000 in men from adults studies	52 (0.0)	[65, 66]
Myasthenia gravis	1.5 per million children per year	27 (0.0)	[67]
Central precocious puberty	9.2 /10,000 in girls in Denmark, 0.9/10,000 in boys in Denmark	220 (0.1)	[31]
Allergic diseases			
Anaphylaxis	0.023% in South Korea	208 (0.1)	[68]
Food allergy	2-10% in South Korea	4,653 (1.2)	[69]
Rheumatic diseases			
Juvenile rheumatoid arthritis	11.9 (95% CI, 10.9-12.9) cases/100,000 in	225 (0.1)	[70]

	children in the United States		

CI, confidence interval; ED, emergency department.

Table S5. Risk for various childhood diseases in breastfed children during the first 4 to 6 months of age compared with formula fed children.

	Unmatched data (n = 374,074)		PS-matched data (n =188,052)		Diseases after 6 months of age		Diseases after 24 months of age	
	N (%)							
	BMF (n=205,807)	FMF (n=168,267)	BMF (n=94,026)	FMF (n=94,026)	RR	95% CI	RR	95% CI
Certainly defined childhood diseases								
Neurologic diseases								
Febrile convulsion	12,011 (5.8)	11,580 (6.9)	5,515 (5.9)	6,264 (6.7)	**0.88**	**0.85 to 0.91**	**0.90**	**0.87 to 0.94**
Epilepsy	628 (0.3)	618 (0.4)	279 (0.3)	285 (0.3)	1.00	0.83 to 1.15	1.00	0.71 to 1.23
Attention deficit hyperactivity disorder	1,525 (0.7)	1,686 (1.0)	677 (0.7)	961 (1.0)	**NA**		**0.79**	**0.71 to 0.87**
Autism spectrum disorder	268 (0.1)	338 (0.2)	126 (0.1)	177 (0.2)	**NA**		**0.72**	**0.57 to 0.89**
Infectious diseases								
Pneumonia	49,719 (24.2)	47,624 (28.3)	22,484 (23.9)	26,080 (27.7)	**0.86**	**0.85 to 0.88**	**0.87**	**0.86 to 0.89**
Acute bronchiolitis	12,079 (5.9)	13,081 (7.8)	5,505 (5.9)	6,994 (7.4)	**0.79**	**0.76 to 0.81**	**0.83**	**0.78 to 0.88**
Tonsillectomy or adenoidectomy	7,018 (3.4)	6,134 (3.7)	3,193 (3.4)	3,436 (3.7)	**0.94**	**0.89 to 0.98**	**0.93**	**0.89 to 0.97**
Gastrointestinal diseases								
Hypertrophic pyloric stenosis	34 (0.02)	151 (0.09)	18 (0.02)	75 (0.08)	**0.27**	**0.14 to 0.40**	NA	
Intussusception	903 (0.4)	824 (0.5)	424 (0.5)	461 (0.5)	0.92	0.81 to 1.05	0.92	0.79 to 1.07
Heart diseases								
Kawasaki disease	1220 (0.6)	1031 (0.6)	575 (0.6)	574 (0.6)	1.00	0.89 to 1.12	0.82	0.34 to 1.30
Hemato-oncologic diseases								
Malignancy	415 (0.2)	358 (0.2)	191 (0.2)	178 (0.2)	1.07	0.88 to 1.32	1.01	0.81 to 1.26
Allergic diseases								
Asthma	5,884 (2.9)	6,120 (3.6)	2,656 (2.8)	3,149 (3.4)	**0.84**	**0.80 to 0.89**	**0.87**	**0.82 to 0.93**
Atopic dermatitis	23,422 (11.4)	16,216 (9.6)	9,983 (10.6)	8,838 (9.4)	**1.13**	**1.10 to 1.16**	**1.10**	**1.06 to 1.14**
Chronic urticaria	1,383 (0.7)	1058 (0.6)	632 (0.7)	561 (0.6)	**1.13**	**1.01 to 1.26**	**1.14**	**1.01 to 1.28**
Other disease**s**								
Alopecia areata	32 (0.02)	43 (0.03)	10 (0.01)	27 (0.03)	**0.47**	**0.18 to 0.77**	0.97	0.88 to 1.08

ICD-10 code-based childhood diseases								
Gastrointestinal diseases								
Irritable bowel syndrome	4,481 (2.18)	4,247 (2.52)	2,006 (2.13)	2,354 (2.50)	**0.85**	**0.80 to 0.90**	**0.85**	**0.79 to 0.93**
Acute pancreatitis	27 (0.01)	20 (0.01)	14 (0.01)	10 (0.01)	1.40	0.62 to 3.15	1.40	0.63 to 3.15
Chronic viral (B, C) hepatitis	28 (0.01)	20 (0.01)	94 (0.10)	156 (0.17)	0.55	0.26 to 1.15	0.83	0.25 to 2.73
Cardiovascular diseases								
Arrhythmia	76 (0.08)	59 (0.04)	36 (0.04)	22 (0.02)	1.64	0.96 to 2.78	1.13	0.57 to 2.21
Acute myocarditis	11 (0.01)	9 (0.01)	4 (0.00)	2 (0.00)	2.00	0.37 to 10.92	1.50	0.26 to 8.98
Hemato-oncologic diseases								
Idiopathic thrombo-cytopenic purpura	485 (0.52)	400 (0.24)	236 (0.25)	205 (0.22)	1.15	0.96 to 1.39	1.03	0.82 to 1.29
Iron deficiency anemia	26,471(28.15)	9,723 (5.78)	11,887(12.64)	5,220 (5.55)	**2.28**	**2.21 to 2.35**	**1.47**	**1.40 to 1.53**
Hemolytic anemia	124 (0.06)	71 (0.04)	51 (0.05)	39 (0.04)	1.31	0.86 to 1.99	0.95	0.52 to 1.76
Kidney diseases								
Nephrotic syndrome	35 (0.02)	33 (0.02)	20 (0.02)	21 (0.02)	0.95	0.52 to 1.76	1.0	0.53 to 1.89
Chronic kidney diseases	4 (0.00)	3 (0.00)	3 (0.00)	2 (0.00)	1.5	0.26 to 8.98	1.5	0.26 to 8.98
Henoch-Schönlein purpura	1308 (1.39)	1138 (0.68)	590 (0.63)	621 (0.66)	0.95	0.85 to 1.06	NA	
Endocrine diseases								
Congenital hypothyroidism	200 (0.10)	136 (0.08)	210 (0.22)	185 (0.20)	1.33	0.98 to 1.82	**1.49**	**1.06 to 2.10**
Goiter	343 (0.36)	267 (0.16)	92 (0.10)	91 (0.10)	0.93	0.74 to 1.16	0.94	0.75 to 1.27
Hashimoto’s disease	27 (0.01)	25 (0.01)	14 (0.01)	11 (0.01)	1.27	0.58 to 2.80	1.27	0.58 to 2.80
Myasthenia gravis	17 (0.01)	10 (0.01)	8 (0.01)	6 (0.01)	1.33	0.47 to 3.84	1.34	0.47 to 3.84
Central precocious puberty	115 (0.12)	105 (0.06)	41 (0.04)	51 (0.05)	0.80	0.53 to 1.21	0.43	0.11 to 1.76
Allergic diseases								
Anaphylaxis	125 (0.06)	83 (0.05)	153 (0.16)	88 (0.09)	1.44	0.97 to 2.14	1.29	0.84 to 1.97
Food allergy	2754 (1.34)	1899 (1.13)	1231 (1.31)	1085 (1.15)	**1.13**	**1.05 to 1.23**	1.59	0.87 to 2.91
Rheumatic diseases								
Juvenile rheumatoid arthritis	118 (0.06)	107 (0.06)	58 (0.06)	65 (0.07)	0.87	0.61 to 1.23	0.85	0.60 to 1.21

NA, not applicable; RR, risk ratio; N, number.

Table S6. The risk of all-cause hospitalization and intensive care unit admission during childhood in children breastfed in their first 4 to 6 months of age, when formula fed infants during the first 4 to 6 months of age were considered reference group

Variables	Feeding types	Unmatched, N (%)	Matched, N (%)	RR	95% CI
All-cause hospitalization					
> 6 months of age	Formula feeding	98,057 (58.3)	54,079 (57.5)	Ref.	
	Breastfeeding	109,841 (53.4)	50,102 (53.3)	**0.93**	**0.92 to 0.94**
> 24 months of age	Formula feeding	76,682 (45.6)	42,339 (45.0)	Ref.	
	Breastfeeding	85,091 (41.4)	38,891 (41.4)	**0.93**	**0.91 to 0.93**
All-cause ICU admission					
> 6 months of age	Formula feeding	876 (0.52)	480 (0.5)	Ref.	
	Breastfeeding	849 (0.41)	375 (0.4)	**0.78**	**0.68 to 0.89**
> 24 months of age	Formula feeding	492 (0.29)	259 (0.3)	Ref.	
	Breastfeeding	509 (0.25)	221 (0.2)	0.85	0.71 to 1.02
All-cause death					
> 6 months of age	Formula feeding	196 (0.12)	97 (0.1)	Ref.	
	Breastfeeding	230 (0.11)	110 (0.1)	1.17	0.86 to 1.49
> 24 months of age	Formula feeding	145 (0.09)	69 (0.07)	Ref.	
	Breastfeeding	158 (0.08)	75 (0.08)	1.15	0.78 to 1.51
Number of all-cause hospitalizations					
0	Formula feeding	70,210 (41.7)	39,947 (42.5)		
	Breastfeeding	95,966 (46.6)	43,924 (46.7)	Ref.	
1-2	Formula feeding	56,348 (33.5)	31,460 (33.5)		
	Breastfeeding	67,251 (32.7)	30,707 (32.7)	**0.89**	**0.87 to 0.91**
3-5	Formula feeding	27,068 (16.1)	14,877(15.8)		
	Breastfeeding	29,043 (14.1)	13,283 (14.1)	**0.81**	**0.79 to 0.83**
≥ 6	Formula feeding	14,641 (8.7)	7,742 (8.2)		
	Breastfeeding	13,547 (6.6)	6,112 (6.5)	**0.72**	**0.69 to 0.74**

Values in bold are considered to be significant.

CI, confidence interval; ICU, intensive care unit; N, number; Ref. reference

Table S7. The risk of all-cause hospitalization and intensive care unit admission after 24 months of age and their growth in children stratified into the duration of exclusive breastfeeding (first 4 to 6 months of age or over 6 months of age), when formula fed infants during the first 4 to 6 months of age were considered reference group

Exclusive BMF duration	Matched, N (%)	RR	95% CI
All-cause hospitalization after 24 months of age			
Formula feeding	26,446 (45.5)	Ref.	
4~6 months of age	19,715 (41.5)	**0.849**	**0.828 to 0.870**
≥ 6 months of age	4,561 (42.5)	**0.886**	**0.849 to 0.923**
All-cause ICU admission after 24 months of age			
Formula feeding	159 (0.3)	Ref.	
4~6 months of age	112 (0.2)	0.861	0.676 to 1.097
≥ 6 months of age	23 (0.2)	0.783	0.506 to 1.213
All-cause death after 24 months of age			
Formula feeding	42 (0.1)	Ref.	
4~6 months of age	42 (0.1)	1.223	0.797 to 1.876
≥ 6 months of age	9 (0.1)	1.161	0.565 to 2.385
Overweight			
Formula feeding	10,250 (18.7)	Ref.	
4~6 months of age	7,863 (17.5)	**-0.068**	**-0.095 to 0.042**
≥ 6 months of age	1,938 (19.1)	0.019	-0.025 to 0.063
Obesity			
Formula feeding	3,997 (7.3)	Ref.	
4~6 months of age	2,828 (6.3)	**-0.149**	**-0.196 to -0.102**
≥ 6 months of age	719 (7.1)	-0.031	-0.108 to 0.046
-1.64 < HFA z score ≤ -1.03			
Formula feeding	4,345 (7.9)	Ref.	
4~6 months of age	3,218 (7.2)	**-0.103**	**-0.147 to -0.059**
≥ 6 months of age	800 (7.9)	-0.008	-0.080 to 0.065
Short stature			
Formula feeding	1,076 (2.0)	Ref.	
4~6 months of age	702 (1.6)	**-0.230**	**-0.324 to -0.136**
≥ 6 months of age	190 (1.9)	-0.050	-0.202 to 0.103

RR, risk ratio; CI, confidence interval; ICU, intensive care unit; N, number; HFA, height for age; Ref., reference; BMI, body mass index

Overweight was defined as BMI z score ≥ 1.03 and obesity as BMI z score ≥ 1.64.

Short stature as HFA z score was defined as height for age z score ≤ -1.63.

Values in bold are considered to be significant.

Table S8. Gender differences of risk of childhood diseases in children who were breastfed in the first 4 to 6 months of age with children fed formula milk as the reference group

Certainly defined childhood diseases	**Risk Difference**			
	**Boys**		**Girls**	
	Estimation	95% CI	Estimation	95% CI
Febrile convulsion	**-0.13**	**-0.18 to -0.09**	**-0.12**	**-0.17 to -0.07**
Epilepsy	-0.04	-0.27 to 0.20	-0.01	-0.24 to 0.22
Attention deficit hyperactivity disorder	**-0.17**	**-0.29 to -0.04**	**-0.39**	**-0.70 to -0.09**
Autism spectrum disorder	**-0.39**	**-0.60 to -0.19**	-0.37	-0.81 to 0.08
Pneumonia	**-0.15**	**-0.17 to -0.13**	**-0.15**	**-0.17 to -0.12**
Acute bronchiolitis	**-0.24**	**-0.29 to -0.20**	**-0.23**	**-0.29 to -0.18**
Tonsillectomy or adenoidectomy	**-0.07**	**-0.13 to -0.01**	-0.07	-0.15 to 0.01
Hypertrophic pyloric stenosis with pyloromyotomy	**-1.27**	**-1.83 to -0.72**	**-2.16**	**-3.62 to -0.6**
Intussusception requiring air or manual reduction	-0.07	-0.23 to 0.09	-0.10	-0.32 to 0.12
Kawasaki disease with IVIG and aspirin	-0.08	-0.24 to 0.07	0.12	-0.06 to 0.29
Malignancy	0.04	-0.24 to 0.31	0.11	-0.19 to 0.41
Asthma exacerbation requiring hospitalization	**-0.20**	**-0.27 to -0.13**	**-0.12**	**-0.20 to -0.04**
Atopic dermatitis	**0.14**	**0.09 to 0.17**	**0.11**	**0.07 to 0.15**
Chronic urticaria	0.11	-0.04 to 0.25	0.14	-0.04 to 0.32
Alopecia areata	-0.68	-1.59 to 0.23	**-1.48**	**-2.74 to -0.23**

Values in bold are considered to be significant.

Table S9. Risk of all-cause admission in children who were breastfed in the first 4-6 months of age when stratified by gender

	Variables	Unmatched, N (%)	Matched, N (%)	Estimate	Wald 95% CI
Boys	All-cause hospitalization after birth				
	Formula feeding	61879 (69.08)	33114 (69.01)	Ref.	
	Breastfeeding	64499 (63.48)	30320 (63.19)	**-0.07**	**-0.08 to -0.06**
	All-cause hospitalization after 6 months of age				
	Formula feeding	54255 (60.57)	29172 (60.79)	Ref.	
	Breastfeeding	56462 (55.57)	26558 (55.35)	**-0.08**	**-0.09 to -0.07**
Girls	All-cause hospitalization after birth				
	Formula feeding	49820 (63.31)	28068 (58.49)	Ref.	
	Breastfeeding	60532 (58.09)	26682 (55.60)	**-0.07**	**-0.08 to -0.06**
	All-cause hospitalization after 6 months of age				
	Formula feeding	43525 (55.31)	24755 (51.59)	Ref.	
	Breastfeeding	53063 (50.93)	23409 (48.78)	**-0.07**	**-0.08 to -0.06**

Ref, reference; CI, confidence interval; N, number.
Values in bold are considered to be significant.

Table S10. Effect of breastfeeding during the first 4 to 6 months of age on body weight and height during childhood

Variables, N (%)		Feeding types	All data	Matched data	Estimate	Wald 95% CI
All	Body weight					
	Overweight	FMF	40,572 (19.7)	21,950 (23.3)	Ref.	
		BMF	47,542 (28.3)	22,670 (24.1)	**-0.03**	**-0.05 to -0.01**
	Obesity	FMF	16,291 (7.9)	8,142 (8.7)	Ref.	
		BMF	17,532 (10.4)	9,111 (9.7)	**-0.11**	**-0.14 to -0.08**
	Height					
	-1.63 < HFA z score ≤ -1.03	FMF	11,929 (7.1)	6,846 (7.3)	Ref.	
		BMF	15,521 (7.5)	6,364 (6.8)	**-0.08**	**-0.11 to -0.05**
	Short stature	FMF	3,014 (1.8)	1,431 (1.5)	Ref.	
		BMF	3,014 (1.8)	1,715 (1.8)	**-0.19**	**-0.26 to -0.12**
Boys	Body weight					
	Overweight	FMF	21,520 (24.03)	11,745 (24.48)	Ref.	
		BMF	23,334 (22.96)	11,147 (23.23)	**-0.10**	**-0.13 to -0.07**
	Obesity	FMF	8,598 (9.60)	4,703 (9.80)	Ref	
		BMF	8,495 (8.36)	4,110 (8.57)	**-0.18**	**-0.23 to -0.14**
	Height					
	-1.63 < HFA z score ≤ -1.03	FMF	6,259 (7.0)	3,514 (7.2)	Ref.	
		BMF	7,565 (7.5)	3,273 (6.8)	**-0.06**	**-0.11 to -0.02**
	Short stature	FMF	1,537 (1.7)	871 (1.8)	Ref.	
		BMF	1,689 (1.7)	707 (1.5)	**-0.20**	**-0.30 to -0.10**
Girls	Body weight					
	Overweight	FMF	19,052 (24.21)	10,925 (22.77)	Ref.	
		BMF	24,208 (23.23)	10,803 (22.51)	**-0.07**	**-0.10 to -0.04**
	Obesity	FMF	7,693 (9.78)	4,408 (9.19)	Ref.	
		BMF	9,037 (8.67)	3,727 (7.77)	**-0.16**	**-0.21 to -0.12**
	Body height					
	-1.63 < HFA z score ≤ -1.03	FMF	5,670 (7.2)	3,332 (7.4)	Ref.	
		BMF	7,956 (7.6)	3,091 (6.7)	**-0.10**	**-0.15 to -0.05**
	Short stature	FMF	1,477 (1.9)	844 (1.9)	Ref.	
		BMF	1,907 (1.8)	724 (1.6)	**-0.18**	**-0.28 to -0.08**

N, number; CI, confidence interval; Ref. reference; BMI, body mass index; HFA, height for age; FMF, formula milk feeding; BMF, breastmilk feeding.
Values in bold are considered to be significant.
Overweight was defined as BMI z score ≥ 1.03 and obesity as BMI z score ≥ 1.64.
Short stature as HFA z score was defined as height for age z score ≤ -1.63.

Table S11. Comparisons of the results of the previous studies on association between breastfeeding and ICD-10 code-based childhood diseases

**Childhood diseases**	**Previous studies**	**Present study**
Gastrointestinal diseases		
Irritable bowel syndrome	NA	↓
Acute pancreatitis	NA	No association
Chronic viral (B, C) hepatitis	No association[71]	No association
Cardiovascular diseases		
Arrhythmia	NA	No association
Acute myocarditis	NA	No association
Hemato-oncologic diseases		
Idiopathic thrombocytopenic purpura	NA	No association
Iron deficiency anemia	↑[72]	↑
Hemolytic anemia	NA	No association
Kidney diseases		
Nephrotic syndrome	NA	No association
Chronic kidney diseases	↓[73]	No association
Henoch-schönlein purpura	↓[74]	No association
Endocrine diseases		
Congenital hypothyroidism	No association[75]	No association
Goiter	NA	No association
Hashimoto’s disease	NA	No association
Myasthenia gravis	NA	No association
Central precocious puberty	↓[76]	No association
Allergic diseases		
Anaphylaxis	NA	No association
Food allergy	Controversial [77, 78]	↑
Rheumatic diseases		
Juvenile rheumatoid arthritis	↓[79]	No association

↓ breastfeeding shows the protective effects on each disease

↑ breastfeeding shows the increased associations with each disease.

NA: not applicable –no research has been identified.

References

1. Han DH, Kim SY, Lee NM, et al. Seasonal distribution of febrile seizure and the relationship with respiratory and enteric viruses in Korean children based on nationwide registry data. *Seizure*. Dec 2019;73:9-13. doi:10.1016/j.seizure.2019.10.008
2. Schubert-Bast S, Zollner JP, Ansorge S, et al. Burden and epidemiology of status epilepticus in infants, children, and adolescents: A population-based study on German health insurance data. *Epilepsia*. May 2019;60(5):911-920. doi:10.1111/epi.14729
3. Perez-Crespo L, Canals-Sans J, Suades-Gonzalez E, Guxens M. Temporal trends and geographical variability of the prevalence and incidence of attention deficit/hyperactivity disorder diagnoses among children in Catalonia, Spain. *Sci Rep*. Apr 14 2020;10(1):6397. doi:10.1038/s41598-020-63342-8
4. Rackauskaite G, Bilenberg N, Uldall P, Bech BH, Ostergaard J. Prevalence of mental disorders in children and adolescents with cerebral palsy: Danish nationwide follow-up study. *Eur J Paediatr Neurol*. Jul 2020;27:98-103. doi:10.1016/j.ejpn.2020.03.004
5. Ruuskanen O, Lahti E, Jennings LC, Murdoch DR. Viral pneumonia. *Lancet*. Apr 9 2011;377(9773):1264-75. doi:10.1016/S0140-6736(10)61459-6
6. Zurita-Cruz JN, Gutierrez-Gonzalez A, Manuel-Apolinar L, et al. Hospitalizations for viral respiratory infections in children under 2 years of age: epidemiology and in-hospital complications. *BMC Pediatr*. Jun 9 2020;20(1):285. doi:10.1186/s12887-020-02186-7
7. Safford SD, Pietrobon R, Safford KM, Martins H, Skinner MA, Rice HE. A study of 11,003 patients with hypertrophic pyloric stenosis and the association between surgeon and hospital volume and outcomes. *J Pediatr Surg*. Jun 2005;40(6):967-72; discussion 972-3. doi:10.1016/j.jpedsurg.2005.03.011
8. Ducharme R, Benchimol EI, Deeks SL, Hawken S, Fergusson DA, Wilson K. Validation of diagnostic codes for intussusception and quantification of childhood intussusception incidence in Ontario, Canada: a population-based study. *J Pediatr*. Oct 2013;163(4):1073-9 e3. doi:10.1016/j.jpeds.2013.05.034
9. Huang YH, Lin KM, Ho SC, Yan JH, Lo MH, Kuo HC. Increased Incidence of Kawasaki Disease in Taiwan in Recent Years: A 15 Years Nationwide Population-Based Cohort Study. *Front Pediatr*. 2019;7:121. doi:10.3389/fped.2019.00121
10. Okubo Y, Nochioka K, Hataya H, Sakakibara H, Terakawa T, Testa M. Burden of Obesity on Pediatric Inpatients with Acute Asthma Exacerbation in the United States. *J Allergy Clin Immunol Pract*. Nov - Dec 2016;4(6):1227-1231. doi:10.1016/j.jaip.2016.06.004
11. Gerner T, Haugaard JH, Vestergaard C, et al. Healthcare utilization in Danish children with atopic dermatitis and parental topical corticosteroid phobia. *Pediatr Allergy Immunol*. Oct 12 2020;doi:10.1111/pai.13394
12. Kim BR, Yang S, Choi JW, Choi CW, Youn SW. Epidemiology and comorbidities of patients with chronic urticaria in Korea: A nationwide population-based study. *J Dermatol*. Jan

2018;45(1):10-16. doi:10.1111/1346-8138.14075
13. Lee S, Lee YB, Kim BJ, Bae S, Lee WS. All-Cause and Cause-Specific Mortality Risks Associated With Alopecia Areata: A Korean Nationwide Population-Based Study. *JAMA Dermatol*. May 29 2019;doi:10.1001/jamadermatol.2019.0629
14. Hsu YC, Yang HY, Huang WT, Chen SC, Lee HS. Use of antidepressants and risks of restless legs syndrome in patients with irritable bowel syndrome: A population-based cohort study. *PLoS One*. 2019;14(8):e0220641. doi:10.1371/journal.pone.0220641
15. Thavamani A, Umapathi KK, Roy A, Krishna SG. The increasing prevalence and adverse impact of morbid obesity in paediatric acute pancreatitis. *Pediatr Obes*. Aug 2020;15(8):e12643. doi:10.1111/ijpo.12643
16. Bolourani S, Diao L, Thompson DA, et al. Risk Factors for Early Readmission After Acute Pancreatitis: Importance of Timely Interventions. *J Surg Res*. Aug 2020;252:96-106. doi:10.1016/j.jss.2020.03.003
17. Chung JW, Choi HY, Ki M, Jang ES, Jeong SH. Comorbidities and Prescribed Medications in Korean Patients with Chronic Hepatitis C: A Nationwide, Population-Based Study. *Gut Liver*. Jul 6 2020;doi:10.5009/gnl19387
18. Mardh O, Quinten C, Amato-Gauci AJ, Duffell E. Mortality from liver diseases attributable to hepatitis B and C in the EU/EEA - descriptive analysis and estimation of 2015 baseline. *Infect Dis (Lond)*. Sep 2020;52(9):625-637. doi:10.1080/23744235.2020.1766104
19. Clausen H, Theophilos T, Jackno K, Babl FE. Paediatric arrhythmias in the emergency department. *Emerg Med J*. Sep 2012;29(9):732-7. doi:10.1136/emermed-2011-200242
20. Idowu RT, Carnahan R, Sathe NA, McPheeters ML. A systematic review of validated methods to capture myopericarditis using administrative or claims data. *Vaccine*. Dec 30 2013;31 Suppl 10:K34-40. doi:10.1016/j.vaccine.2013.08.074
21. Bhatt NS, Bhatt P, Donda K, et al. Temporal trends of splenectomy in pediatric hospitalizations with immune thrombocytopenia. *Pediatr Blood Cancer*. Jul 2018;65(7):e27072. doi:10.1002/pbc.27072
22. Lin J, Zhang X, Li X, et al. Cost of Bleeding-related Episodes in Adult Patients With Primary Immune Thrombocytopenia: A Population-based Retrospective Cohort Study of Administrative Claims Data for Commercial Payers in the United States. *Clin Ther*. Mar 2017;39(3):603-609 e1. doi:10.1016/j.clinthera.2017.01.023
23. Hansen DL, Overgaard UM, Pedersen L, Frederiksen H. Positive predictive value of diagnosis coding for hemolytic anemias in the Danish National Patient Register. *Clin Epidemiol*. 2016;8:241-52. doi:10.2147/CLEP.S93643
24. Chang JW, Tsai HL, Yang LY, Chen TJ. Epidemiology and predictors of end-stage renal disease in Taiwanese children with idiopathic nephrotic syndrome. *J Epidemiol*. 2012;22(6):517-22. doi:10.2188/jea.JE20120033
25. Tan XW, Xie Y, Lew JK, Lee PSS, Lee ES. Patterns of patients with multiple chronic

conditions in primary care: A cross-sectional study. *PLoS One*. 2020;15(8):e0238353. doi:10.1371/journal.pone.0238353
26. Yagnik P, Jain A, Amponsah JK, et al. National Trends in the Epidemiology and Resource Use for Henoch-Schonlein Purpura (IgA Vasculitis) Hospitalizations in the United States From 2006 to 2014. *Hosp Pediatr*. Nov 2019;9(11):888-896. doi:10.1542/hpeds.2019-0131
27. Ooki S. Congenital hypothyroidism after assisted reproductive technology in Japan: comparison between multiples and singletons, 2005-2009. *Int J Pediatr Endocrinol*. Feb 12 2013;2013(1):5. doi:10.1186/1687-9856-2013-5
28. Beck AC, Sugg SL, Weigel RJ, Belding-Schmitt M, Howe JR, Lal G. Racial disparities in comorbid conditions among patients undergoing thyroidectomy for Graves' disease: An ACS-NSQIP analysis. *Am J Surg*. May 21 2020;doi:10.1016/j.amjsurg.2020.05.023
29. Yun JS, Bae JM, Kim KJ, et al. Increased risk of thyroid diseases in patients with systemic lupus erythematosus: A nationwide population-based Study in Korea. *PLoS One*. 2017;12(6):e0179088. doi:10.1371/journal.pone.0179088
30. Hamedani AG, De Lott LB, Deveney T, Moss HE. Validity of International Classification of Diseases Codes for Identifying Neuro-Ophthalmic Disease in Large Data Sets: A Systematic Review. *J Neuroophthalmol*. Dec 2020;40(4):514-519. doi:10.1097/WNO.0000000000000971
31. Brauner EV, Busch AS, Eckert-Lind C, Koch T, Hickey M, Juul A. Trends in the Incidence of Central Precocious Puberty and Normal Variant Puberty Among Children in Denmark, 1998 to 2017. *JAMA Netw Open*. Oct 1 2020;3(10):e2015665. doi:10.1001/jamanetworkopen.2020.15665
32. Robinson LB, Arroyo AC, Faridi MK, Rudders SA, Camargo CA, Jr. Trends in US hospitalizations for anaphylaxis among infants and toddlers: 2006 to 2015. *Ann Allergy Asthma Immunol*. Sep 8 2020;doi:10.1016/j.anai.2020.09.003
33. Tanno LK, Calderon MA, Goldberg BJ, Akdis CA, Papadopoulos NG, Demoly P. Categorization of allergic disorders in the new World Health Organization International Classification of Diseases. *Clin Transl Allergy*. 2014;4:42. doi:10.1186/2045-7022-4-42
34. Shiff NJ, Lix LM, Oen K, et al. Chronic inflammatory arthritis prevalence estimates for children and adolescents in three Canadian provinces. *Rheumatol Int*. Feb 2015;35(2):345-50. doi:10.1007/s00296-014-3085-0
35. Byeon JH, Kim GH, Eun BL. Prevalence, Incidence, and Recurrence of Febrile Seizures in Korean Children Based on National Registry Data. *J Clin Neurol*. Jan 2018;14(1):43-47. doi:10.3988/jcn.2018.14.1.43
36. Aaberg KM, Gunnes N, Bakken IJ, et al. Incidence and Prevalence of Childhood Epilepsy: A Nationwide Cohort Study. *Pediatrics*. May 2017;139(5)doi:10.1542/peds.2016-3908
37. Sayal K, Prasad V, Daley D, Ford T, Coghill D. ADHD in children and young people: prevalence, care pathways, and service provision. *Lancet Psychiatry*. Feb 2018;5(2):175-186. doi:10.1016/S2215-0366(17)30167-0
38. Choi HW, Choi CH, Lim MH, et al. Attention-Deficit/Hyperactivity Disorder Symptom

Characteristics in Korean Elementary School Children: Comparison with US Population. *Psychiatry Investig*. Jun 2019;16(6):425-432. doi:10.30773/pi.2019.03.26
39. Kim YS, Leventhal BL, Koh YJ, et al. Prevalence of autism spectrum disorders in a total population sample. *Am J Psychiatry*. Sep 2011;168(9):904-12. doi:10.1176/appi.ajp.2011.10101532
40. Shin EJ, Kim Y, Jeong JY, Jung YM, Lee MH, Chung EH. The changes of prevalence and etiology of pediatric pneumonia from National Emergency Department Information System in Korea, between 2007 and 2014. *Korean J Pediatr*. Sep 2018;61(9):291-300. doi:10.3345/kjp.2017.06100
41. Fujiogi M, Goto T, Yasunaga H, et al. Trends in Bronchiolitis Hospitalizations in the United States: 2000-2016. *Pediatrics*. Dec 2019;144(6)doi:10.1542/peds.2019-2614
42. Chae SW, Choi G, Hwang KS, Lee DJ, Choi CS, Hwang SJ. The Incidence and Clinical Symptoms of Palatine Tonsillar Hypertrophy in Elementary School Children. *Korean Journal of Otorhinolaryngology-Head and Neck Surgery*. 2000;43(12):1342-1345.
43. Leong MM, Chen SC, Hsieh CS, et al. Epidemiological features of infantile hypertrophic pyloric stenosis in Taiwanese children: a Nation-Wide Analysis of Cases during 1997-2007. *PLoS One*. May 3 2011;6(5):e19404. doi:10.1371/journal.pone.0019404
44. Ratu FT, Reyburn R, Tuivaga E, et al. Epidemiology of intussusception before and after rotavirus vaccine introduction in Fiji. *Sci Rep*. Jul 25 2018;8(1):11194. doi:10.1038/s41598-018-29515-2
45. Lin MT, Wu MH. The global epidemiology of Kawasaki disease: Review and future perspectives. *Glob Cardiol Sci Pract*. Oct 31 2017;2017(3):e201720. doi:10.21542/gcsp.2017.20
46. Steliarova-Foucher E, Colombet M, Ries LAG, et al. International incidence of childhood cancer, 2001-10: a population-based registry study. *Lancet Oncol*. Jun 2017;18(6):719-731. doi:10.1016/S1470-2045(17)30186-9
47. Perry R, Braileanu G, Palmer T, Stevens P. The Economic Burden of Pediatric Asthma in the United States: Literature Review of Current Evidence. *Pharmacoeconomics*. Feb 2019;37(2):155-167. doi:10.1007/s40273-018-0726-2
48. Ahn K. The Prevalence of Atopic Dermatitis in Korean Children. *Allergy Asthma Immunol Res*. Jan 2016;8(1):1-2. doi:10.4168/aair.2016.8.1.1
49. Lee N, Lee JD, Lee HY, Kang DR, Ye YM. Epidemiology of Chronic Urticaria in Korea Using the Korean Health Insurance Database, 2010-2014. *Allergy Asthma Immunol Res*. Sep 2017;9(5):438-445. doi:10.4168/aair.2017.9.5.438
50. Villasante Fricke AC, Miteva M. Epidemiology and burden of alopecia areata: a systematic review. *Clin Cosmet Investig Dermatol*. 2015;8:397-403. doi:10.2147/CCID.S53985
51. Miele E, Simeone D, Marino A, et al. Functional gastrointestinal disorders in children: an Italian prospective survey. *Pediatrics*. Jul 2004;114(1):73-8. doi:10.1542/peds.114.1.73
52. Caplan A, Walker L, Rasquin A. Validation of the pediatric Rome II criteria for functional gastrointestinal disorders using the questionnaire on pediatric gastrointestinal symptoms. *J Pediatr*

Gastroenterol Nutr. Sep 2005;41(3):305-16. doi:10.1097/01.mpg.0000172749.71726.13

53. Sellers ZM, MacIsaac D, Yu H, et al. Nationwide Trends in Acute and Chronic Pancreatitis Among Privately Insured Children and Non-Elderly Adults in the United States, 2007-2014. *Gastroenterology*. Aug 2018;155(2):469-478 e1. doi:10.1053/j.gastro.2018.04.013

54. Indolfi G, Easterbrook P, Dusheiko G, et al. Hepatitis B virus infection in children and adolescents. *Lancet Gastroenterol Hepatol*. Jun 2019;4(6):466-476. doi:10.1016/S2468-1253(19)30042-1

55. Indolfi G, Easterbrook P, Dusheiko G, et al. Hepatitis C virus infection in children and adolescents. *Lancet Gastroenterol Hepatol*. Jun 2019;4(6):477-487. doi:10.1016/S2468-1253(19)30046-9

56. Turner CJ, Wren C. The epidemiology of arrhythmia in infants: a population-based study. *J Paediatr Child Health*. Apr 2013;49(4):278-81. doi:10.1111/jpc.12155

57. Lipshultz SE, Sleeper LA, Towbin JA, et al. The incidence of pediatric cardiomyopathy in two regions of the United States. *N Engl J Med*. Apr 24 2003;348(17):1647-55. doi:10.1056/NEJMoa021715

58. Saeidi S, Jaseb K, Asnafi AA, et al. Immune Thrombocytopenic Purpura in Children and Adults: A Comparative Retrospective Study in IRAN. *Int J Hematol Oncol Stem Cell Res*. Jul 1 2014;8(3):30-6.

59. Zainel A, Osman SRO, Al-Kohji SMS, Selim NA. Iron deficiency, its epidemiological features and feeding practices among infants aged 12 months in Qatar: a cross-sectional study. *BMJ Open*. May 9 2018;8(5):e020271. doi:10.1136/bmjopen-2017-020271

60. Naithani R, Agrawal N, Mahapatra M, Kumar R, Pati HP, Choudhry VP. Autoimmune hemolytic anemia in children. *Pediatr Hematol Oncol*. Jun 2007;24(4):309-15. doi:10.1080/08880010701360783

61. Banh TH, Hussain-Shamsy N, Patel V, et al. Ethnic Differences in Incidence and Outcomes of Childhood Nephrotic Syndrome. *Clin J Am Soc Nephrol*. Oct 7 2016;11(10):1760-1768. doi:10.2215/CJN.00380116

62. Collaboration GBDCKD. Global, regional, and national burden of chronic kidney disease, 1990-2017: a systematic analysis for the Global Burden of Disease Study 2017. *Lancet*. Feb 29 2020;395(10225):709-733. doi:10.1016/S0140-6736(20)30045-3

63. Lei WT, Tsai PL, Chu SH, et al. Incidence and risk factors for recurrent Henoch-Schonlein purpura in children from a 16-year nationwide database. *Pediatr Rheumatol Online J*. Apr 16 2018;16(1):25. doi:10.1186/s12969-018-0247-8

64. Chen CY, Lee KT, Lee CT, Lai WT, Huang YB. Epidemiology and clinical characteristics of congenital hypothyroidism in an Asian population: a nationwide population-based study. *J Epidemiol*. 2013;23(2):85-94. doi:10.2188/jea.je20120113

65. Kwon H, Jung JH, Han KD, et al. Prevalence and Annual Incidence of Thyroid Disease in Korea from 2006 to 2015: A Nationwide Population-Based Cohort Study. *Endocrinol Metab (Seoul)*.

Jun 2018;33(2):260-267. doi:10.3803/EnM.2018.33.2.260
66. Ragusa F, Fallahi P, Elia G, et al. Hashimotos' thyroiditis: Epidemiology, pathogenesis, clinic and therapy. *Best Pract Res Clin Endocrinol Metab*. Dec 2019;33(6):101367. doi:10.1016/j.beem.2019.101367
67. Parr JR, Andrew MJ, Finnis M, Beeson D, Vincent A, Jayawant S. How common is childhood myasthenia? The UK incidence and prevalence of autoimmune and congenital myasthenia. *Archives of disease in childhood*. 2014;99(6):539-542.
68. Cho H, Kwon JW. Prevalence of anaphylaxis and prescription rates of epinephrine auto-injectors in urban and rural areas of Korea. *Korean J Intern Med*. May 2019;34(3):643-650. doi:10.3904/kjim.2018.094
69. Kang SY, Song WJ, Cho SH, Chang YS. Time trends of the prevalence of allergic diseases in Korea: A systematic literature review. *Asia Pac Allergy*. Jan 2018;8(1):e8. doi:10.5415/apallergy.2018.8.e8
70. Harrold LR, Salman C, Shoor S, et al. Incidence and prevalence of juvenile idiopathic arthritis among children in a managed care population, 1996-2009. *J Rheumatol*. Jul 2013;40(7):1218-25. doi:10.3899/jrheum.120661
71. Shi Z, Yang Y, Wang H, et al. Breastfeeding of newborns by mothers carrying hepatitis B virus: a meta-analysis and systematic review. *Arch Pediatr Adolesc Med*. Sep 2011;165(9):837-46. doi:10.1001/archpediatrics.2011.72
72. Chantry CJ, Howard CR, Auinger P. Full breastfeeding duration and risk for iron deficiency in U.S. infants. *Breastfeed Med*. Jun 2007;2(2):63-73. doi:10.1089/bfm.2007.0002
73. Miliku K, Voortman T, Bakker H, Hofman A, Franco OH, Jaddoe VW. Infant Breastfeeding and Kidney Function in School-Aged Children. *Am J Kidney Dis*. Sep 2015;66(3):421-8. doi:10.1053/j.ajkd.2014.12.018
74. Pisacane A, Buffolano W, Grillo G, Gaudiosi C. Infant feeding and Schonlein-Henoch purpura. *Acta Paediatr*. Aug 1992;81(8):630. doi:10.1111/j.1651-2227.1992.tb12315.x
75. Rovet JF. Does breast-feeding protect the hypothyroid infant whose condition is diagnosed by newborn screening? *Am J Dis Child*. Mar 1990;144(3):319-23. doi:10.1001/archpedi.1990.02150270069028
76. Aghaee S, Deardorff J, Greenspan LC, Quesenberry CP, Jr., Kushi LH, Kubo A. Breastfeeding and timing of pubertal onset in girls: a multiethnic population-based prospective cohort study. *BMC Pediatr*. Aug 9 2019;19(1):277. doi:10.1186/s12887-019-1661-x
77. Kusunoki T, Morimoto T, Nishikomori R, et al. Breastfeeding and the prevalence of allergic diseases in schoolchildren: does reverse causation matter? *Pediatric Allergy and Immunology*. 2010;21(1-Part-I):60-66.
78. Mathias JG, Zhang H, Soto-Ramirez N, Karmaus W. The association of infant feeding patterns with food allergy symptoms and food allergy in early childhood. *Int Breastfeed J*. 2019;14:43. doi:10.1186/s13006-019-0241-x

79. Hyrich KL, Baildam E, Pickford H, et al. Influence of past breast feeding on pattern and severity of presentation of juvenile idiopathic arthritis. *Arch Dis Child*. Apr 2016;101(4):348-51. doi:10.1136/archdischild-2014-308117
